# Frailty and the short-term prognosis of patients with intracranial hemorrhage: A meta-analysis

**DOI:** 10.1016/j.jnha.2025.100655

**Published:** 2025-09-09

**Authors:** Caiyun Li, Fei Xia, Yang Ni, Yiwen Liu

**Affiliations:** Department of Critical Care Medicine, West China Hospital, Sichuan University, Chengdu 610041, China

**Keywords:** Frailty, Intracranial hemorrhage, Mortality, Functional outcome, Meta-analysis

## Abstract

**Background:**

Frailty is increasingly recognized as a predictor of poor outcomes in acute neurological conditions. However, its impact on the short-term prognosis of patients with intracranial hemorrhage (ICrH) remains unclear. This meta-analysis aimed to evaluate the association between frailty and short-term mortality and functional outcomes in patients with ICrH.

**Methods:**

A systematic literature search was conducted in PubMed, Embase, and Web of Science from inception to February 10, 2025. Cohort studies evaluating the association between frailty and prognosis in ICrH patients were included. Odds ratios (ORs) with 95% confidence intervals (CIs) were pooled using a random-effects model by incorporating the influence of heterogeneity.

**Results:**

Twelve cohort studies involving 70,664 patients with ICrH were included. Frailty was significantly associated with increased short-term mortality (OR: 1.79, 95% CI: 1.36–2.35, *p* < 0.001; I^2^ = 72%) and poor functional outcome (OR: 1.75, 95% CI: 1.33–2.30, *p* < 0.001; I^2^ = 67%). Subgroup analyses were performed for mortality outcomes and confirmed consistent associations across different patient demographics, frailty assessment tools, and follow-up durations (*p* for subgroup difference all >0.05). Sensitivity analyses by excluding one study at a time showed similar results (*p* all < 0.05), which demonstrated robustness.

**Conclusion:**

Our study suggests that frailty may be associated with an increased risk of short-term mortality and poor functional outcomes in patients with ICrH. These findings highlight the importance of frailty assessment in risk stratification and clinical decision-making for ICrH patients.

## 1 Introduction

Intracranial hemorrhage (ICrH) is a life-threatening cerebrovascular event that accounts for a substantial proportion of stroke-related morbidity and mortality worldwide [1,2]. The two major subtypes, intracerebral hemorrhage (ICH) [3] and subarachnoid hemorrhage (SAH) [4], pose distinct yet equally severe clinical challenges. ICH, characterized by spontaneous bleeding within the brain parenchyma, constitutes approximately 10–15% of all strokes and is associated with high early mortality and long-term disability [5]. SAH, typically caused by the rupture of an intracranial aneurysm, accounts for 5–10% of strokes but carries an especially high risk of mortality and neurological complications, even among patients who survive the initial hemorrhage [6]. Despite advances in neurocritical care and surgical interventions, ICrH remains a leading cause of death and functional dependence, underscoring the need for improved risk stratification to guide early management and optimize patient outcomes [7].

Frailty has emerged as a critical factor influencing clinical prognosis across various medical conditions, particularly in aging populations [8]. Frailty is a multidimensional syndrome characterized by decreased physiological reserves and heightened vulnerability to external stressors, leading to impaired recovery following acute illnesses [9,10]. Unlike chronological age alone, frailty more accurately reflects biological aging and can better predict adverse health outcomes [11]. The clinical significance of frailty extends beyond geriatrics, as it has been increasingly recognized as a prognostic marker in cardiovascular diseases [12], malignancies [13], and surgical outcomes [14]. In the context of neurological disorders, frailty has been linked to worse functional recovery, increased complications, and higher mortality rates, such as in patients with acute ischemic stroke [15,16]. Several mechanisms may explain this association, including chronic systemic inflammation, endothelial dysfunction, impaired neurovascular coupling, and metabolic stress, which contribute to greater susceptibility to cerebrovascular injury and hinder post-hemorrhagic recovery [17,18]. Given the substantial burden of ICrH and the heterogeneous nature of its outcomes, frailty assessment may offer valuable prognostic insights to aid in clinical decision-making.

Despite increasing recognition of frailty as a prognostic factor in neurological conditions, the relationship between frailty and outcomes in ICrH remains inconsistently reported in the literature. Some studies have demonstrated a strong association between frailty and increased mortality, prolonged hospital stays, and poorer functional recovery in patients with ICrH [[19], [20], [21], [22], [23], [24], [25], [26], [27]], while others have yielded non-significant results [[28], [29], [30]], possibly due to variations in frailty assessment methods, study designs, and patient populations. In view of the uncertainty, we performed a meta-analysis in this study aiming to systematically evaluate the association between frailty and short-term prognosis in patients with ICrH, with a focus on all-cause mortality and functional outcomes.

## 2 Methods

The study followed the PRISMA 2020 guidelines [31,32] and the Cochrane Handbook for Systematic Reviews and Meta-Analyses [33] to ensure a rigorous approach in study design, data extraction, statistical evaluation, and result interpretation. The protocol of the meta-analysis has been registered at PROSPERO with the identifier CRD420251026922.

### 2.1 Literature search

To identify relevant studies for this meta-analysis, we conducted a comprehensive search of the PubMed, Embase, and Web of Science databases using a broad range of search terms, including [1] "frailty" OR "frail" and [2] various terms related to cerebral hemorrhage, such as "intracerebral hemorrhage," "brain hemorrhage," "intracranial hemorrhage," "ICH," "cerebral bleeding," "intraparenchymal hemorrhage," "intracranial bleeding," "subarachnoid hemorrhage," "subarachnoid haemorrhage," and "SAH." The search was restricted to studies on human subjects and limited to full-text articles published in English in peer-reviewed journals. Additional relevant studies were identified by manually screening the reference lists of key original and review articles. The literature search covered publications from the inception of each database until February 10, 2025, with detailed search strategies available in Supplementary File S1.

### 2.2 Study selection criteria

The study's inclusion criteria were established using the PICOS framework:

P (Patients): Patients diagnosed with ICrH, including subtypes such as ICH and SAH etc.

I (Exposure): Baseline frailty, as determined by assessment methods reported in the original studies.

C (Comparison): Individuals classified as non-frail at baseline.

O (Outcome): The primary outcome was all-cause mortality, while the secondary outcome was poor functional outcome, both assessed within 12 months of disease onset. The definition of poor functional outcome followed the criteria applied in the original studies.

S (Study Design): Cohort studies, encompassing both prospective and retrospective designs.

Exclusion criteria included reviews, editorials, meta-analyses, preclinical or cross-sectional studies, studies not specifically examining ICrH patients, those not considering frailty as an exposure, and studies lacking relevant outcome data. If multiple studies involved overlapping populations, the one with the largest sample size was selected for analysis.

### 2.3 Study quality evaluation and data extraction

Two independent authors carried out the literature search, study selection, quality assessment, and data extraction. Any discrepancies between reviewers were discussed and resolved through consensus. Study quality was assessed using the predefined domains of the Newcastle–Ottawa Scale (NOS) [34], which evaluates methodological rigor based on cohort representativeness (prospective, consecutively, or randomly selected patients), selection of controls, ascertainment of exposure, confirmation that the outcome was not present at baseline, control for age and sex, adjustment for additional confounders, assessment of outcomes, adequacy of follow-up duration (defined as at least 3 months for functional outcomes), and completeness of follow-up (with <10% loss considered adequate).

### 2.4 Statistical analysis

The relationship between frailty and short-term prognosis in patients with ICrH was assessed using odds ratios (ORs) with corresponding 95% confidence intervals (CIs). When ORs were not directly reported, they were calculated from available 95% CIs or p-values, then logarithmically transformed to stabilize variance. Study heterogeneity was evaluated using the Cochrane Q test and the I² statistic, with an I² value exceeding 50% considered indicative of substantial heterogeneity [35]. To account for variability in patient populations and frailty assessment methods, a random-effects model was applied for meta-analysis [33]. To assess the robustness of the findings, sensitivity analyses were performed by systematically omitting individual studies [33]. For the primary outcome of all-cause mortality, predefined subgroup analyses explored the potential influence of factors such as study location, ICrH subtypes, mean age, male proportion, frailty assessment approach, follow-up duration, and study quality (assessed by NOS scores). Continuous variables were dichotomized using median values for subgroup classification. Publication bias was investigated through funnel plot visualization, assessment of asymmetry, and Egger’s regression test [36]. All statistical analyses were conducted using RevMan (Version 5.1; Cochrane Collaboration, Oxford, UK) and Stata (Version 17.0; StataCorp, College Station, TX, USA).

## 3 Results

### 3.1 Database search and study selection results

**Fig. 1:**
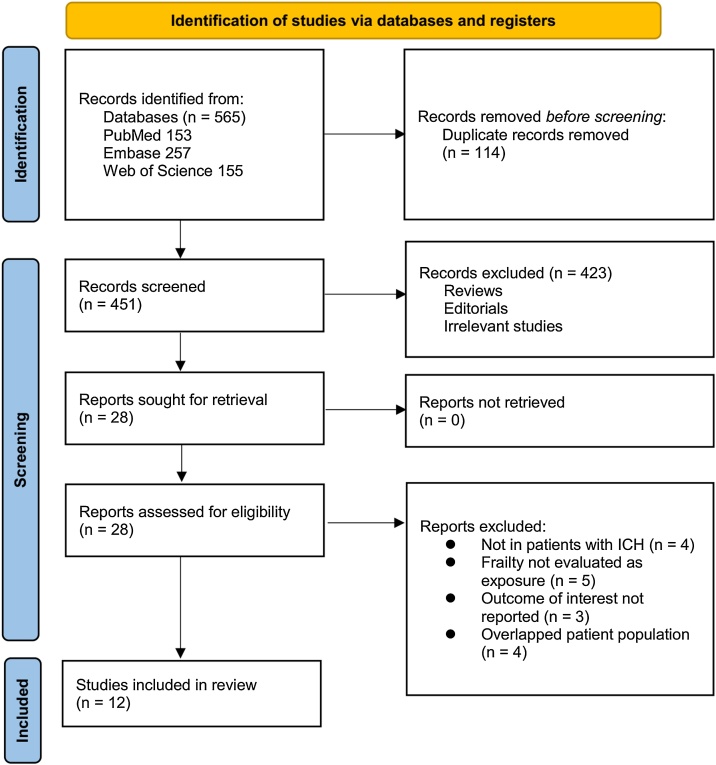
Flowchart of database search and study inclusion.

Fig. 1 provides a detailed overview of the study selection process. A total of 565 potentially relevant records were initially retrieved from the three databases. After eliminating 114 duplicate entries, 423 studies were excluded during title and abstract screening due to irrelevance to the meta-analysis objectives. The remaining 28 full-text articles were independently reviewed by two authors, leading to the exclusion of 16 studies based on predefined criteria outlined in Fig. 1. Ultimately, 12 cohort studies fulfilled the inclusion criteria and were incorporated into the quantitative analysis [[19], [20], [21], [22], [23], [24], [25], [26], [27], [28], [29], [30]].

### 3.2 Overall view of the study characteristics

Table 1 shows the summarized characteristics of the available studies included in the meta-analysis. Overall, 11 retrospective cohorts [[19], [20], [21], [22],[24], [25], [26], [27], [28], [29], [30]] and one prospective cohort [23] were involved in the meta-analysis. These studies were published from 2016 to 2025, and were conducted in China, Japan, the United States, USA, Australia, Singapore, and the United Kingdom. Four studies included patients with ICH [20,23,25,26], seven studies included patients with SAH [19,21,22,24,27,29,30], and another studies included patients with subdural hemorrhage (SDH) [28]. Overall, 70,664 patients with ICrH were included. The mean ages of the included patients were 55.4–76.0 years, and the proportions of men were 19.6–66.5%. Frailty was evaluated by the modified Frailty Index (mFI) in eight studies [20,20,21,22,24,25,[27], [28], [29]], via the Hospital Frailty Risk Score (HFRS) in two studies [23,30], and by the Clinical Frailty Scale (CFS) [26] and the predefined accumulated deficits [19] in another two studies, respectively. The follow-up durations varied from during hospitalization to 12 months after disease onset. The timing of outcome assessment corresponded to the end of the reported follow-up duration, as summarized in Table 1. The outcome of all-cause mortality was reported in 11 studies [20,25,25,26,27,28,29,30], while the outcome of poor functional outcome was reported in six studies [19,20,24,26,29,30]. The poor functional outcome was defined as the modified Rankin Scale (mRS) >2 or >3 in five studies [19,20,26,29,30], and by the National Inpatient Sample – SAH Outcome Measure (NIH-SOM) in another study [24]. Multivariable analyses were performed in all the studies when the association between frailty and outcomes of patients with ICrH was evaluated, with the adjustment of age, sex, volume of hemorrhage, and main treatments etc. to a varying degree. The NOS scores for the included studies ranged from seven to nine, indicating high study quality (Table 2). Although most of the included studies were retrospective in design and varied in sample size, they fulfilled the majority of these NOS criteria—particularly regarding confounder control through multivariable adjustment, clearly defined outcome measures, and acceptable follow-up completeness—resulting in high total quality scores.

**Table 1:** Characteristics of the included studies.

Study	Country	Design	Diagnosis	Sample size	Mean age (years)	Men (%)	Methods for evaluating frailty	Number of patients with frailty	Follow-up duration (months)	Outcomes reported	Variables adjusted
Yue 2016	China	RC	aSAH	109	NR (>60 years)	28.4	Predefined accumulated deficits	50	3	Poor functional outcome (mRS: 3−6)	Age, sex, comorbidities, Hunt and Hess scale, feature of aneurysm, and treatments
Imaoka 2018	Japan	RC	ICH	156	66	59	mFI	NR	6	All-cause mortality and poor functional outcome (mRS: 4−6)	Age, sex, concurrent treatment, GCS at admission, IVH, and hemorrhage volume
McIntyre 2019	USA	RC	aSAH	217	57.6	34.6	mFI	57	Inhospital	All-cause mortality	Age, sex, race, BMI, smoking, Hunt and Hess scale, Fisher Score, feature of aneurysm, and treatments
Zhang 2020	Australia	PC	ICH	2098	76	52	HFRS	561	6	All-cause mortality	Age, sex, comorbidities, SES, and ability to walk on admission
Rawanduzy 2020	USA	RC	SDH	167	63.4	66.5	mFI	71	Inhospital	All-cause mortality	Age, sex, smoking, comorbidities, GCS at admission, acute subdural hematoma thickness, initial midline shift, concurrent anticoagulant/antiplatelet use, and craniotomy
McIntyre 2020	USA	RC	Angiogram negative SAH	75	55.4	44	mFI	32	Inhospital	All-cause mortality	Age, sex, race, BMI, smoking, Hunt and Hess scale, Fisher Score, dyslipidemia, and treatments
Lim 2022	Singapore	RC	aSAH	51	58.8	19.6	mFI	31	12	All-cause mortality and poor functional outcome (mRS: 4−6)	Age, sex, smoking, GCS at admission, modified Fisher Score, and treatments
Dicpinigaitis 2022	USA	RC	aSAH	64102	55.4	31.5	mFI	23141	Inhospital	All-cause mortality and poor functional outcome (NIS-SOM)	Age, sex, race, NIS-SSS, and endovascular treatments
Lewis 2024	UK	RC	ICH	116	77.2	46.6	mFI	NR	1	All-cause mortality	Age, sex, hemorrhage volume, and neurosurgical treatments
Ong 2024	Singapore	RC	ICH	1091	65	60.8	CFS	540	3	All-cause mortality and poor functional outcome (mRS: 3−6)	Age, sex, race, comorbidities, hemorrhage volume, and treatments
Yamamoto 2024	Japan	RC	aSAH	1343	NR	28.2	HFRS	342	Inhospital	All-cause mortality and poor functional outcome (mRS: 3−6)	Age, sex, BMI, aneurysm location, neurosurgical procedure on admission, route of admission, mRS before onset, BI score at admission, JCS score at admission, ICU management on admission, inpatient rehabilitation services received, number of beds, and year of admission
AbuHasan 2025	USA	RC	Non-traumatic SAH	1139	NR	33.7	mFI	582	1	All-cause mortality	Age, sex, race, obesity, smoking, obesity, and ASA class

ASA, American Society of Anesthesiologists; aSAH, aneurysmal subarachnoid hemorrhage; BI, Barthel index; BMI, body mass index; CFS, Clinical Frailty Scale; GCS, Glasgow coma scale; HFRS, Hospital Frailty Risk Score; ICU, intensive care unit; ICH, intracerebral hemorrhage; IVH, intraventricular hemorrhage; JCS, Japan Coma Scale; mFI, modified Frailty Index; mRS, modified Rankin Scale; NIS-SOM, National Inpatient Sample – SAH Outcome Measure; NIS-SSS, National Inpatient Sample - Severity of Illness Score; NR, not reported; PC, prospective cohort; RC, retrospective cohort; SAH, subarachnoid hemorrhage; SDH, subdural hemorrhage; SES, socioeconomic status;

**Table 2:** Study quality evaluation via the Newcastle-Ottawa Scale.

Study	Representativeness of the exposed cohort	Selection of the non-exposed cohort	Ascertainment of exposure	Outcome not present at baseline	Control for age and sex	Control for other confounding factors	Assessment of outcome	Enough long follow-up duration	Adequacy of follow-up of cohorts	Total
Yue 2016	0	1	1	1	1	1	1	1	1	8
Imaoka 2018	1	1	1	1	1	1	1	1	1	9
McIntyre 2019	1	1	1	1	1	1	1	0	1	8
Zhang 2020	1	1	1	1	1	1	1	1	1	9
Rawanduzy 2020	0	1	1	1	1	1	1	0	1	7
McIntyre 2020	1	1	1	1	1	1	1	0	1	8
Lim 2022	0	1	1	1	1	1	1	1	1	8
Dicpinigaitis 2022	0	1	1	1	1	1	1	0	1	7
Lewis 2024	1	1	1	1	1	1	1	0	1	8
Ong 2024	1	1	1	1	1	1	1	1	1	9
Yamamoto 2024	0	1	1	1	1	1	1	0	1	7
AbuHasan 2025	0	1	1	1	1	1	1	0	1	7

### 3.3 Frailty and the short-term prognosis

**Fig. 2:**
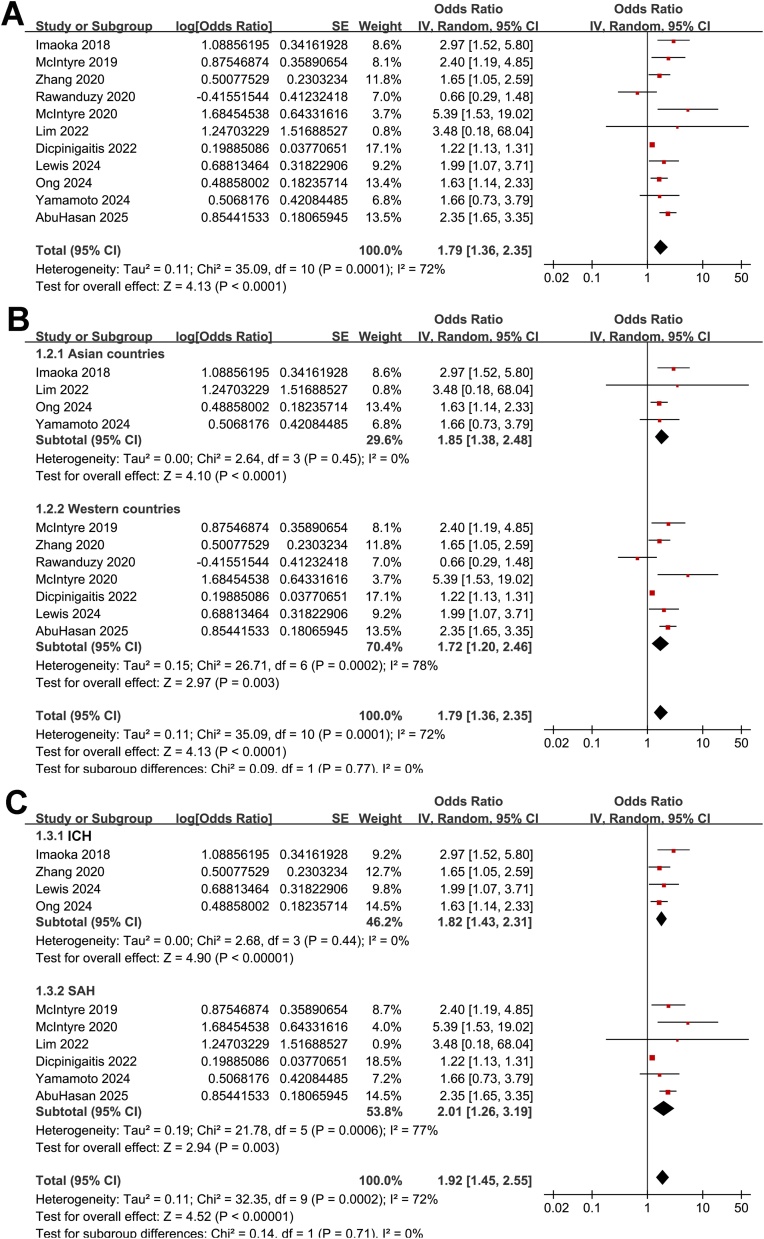
Forest plots for the meta-analysis of the association between frailty and short-term mortality of patients with ICrH. A, overall meta-analysis; B, subgroup analysis according to study country; and C, subgroup analysis according to the subtype of ICrH; All estimates are adjusted ORs derived from multivariable analyses.

**Fig. 3:**
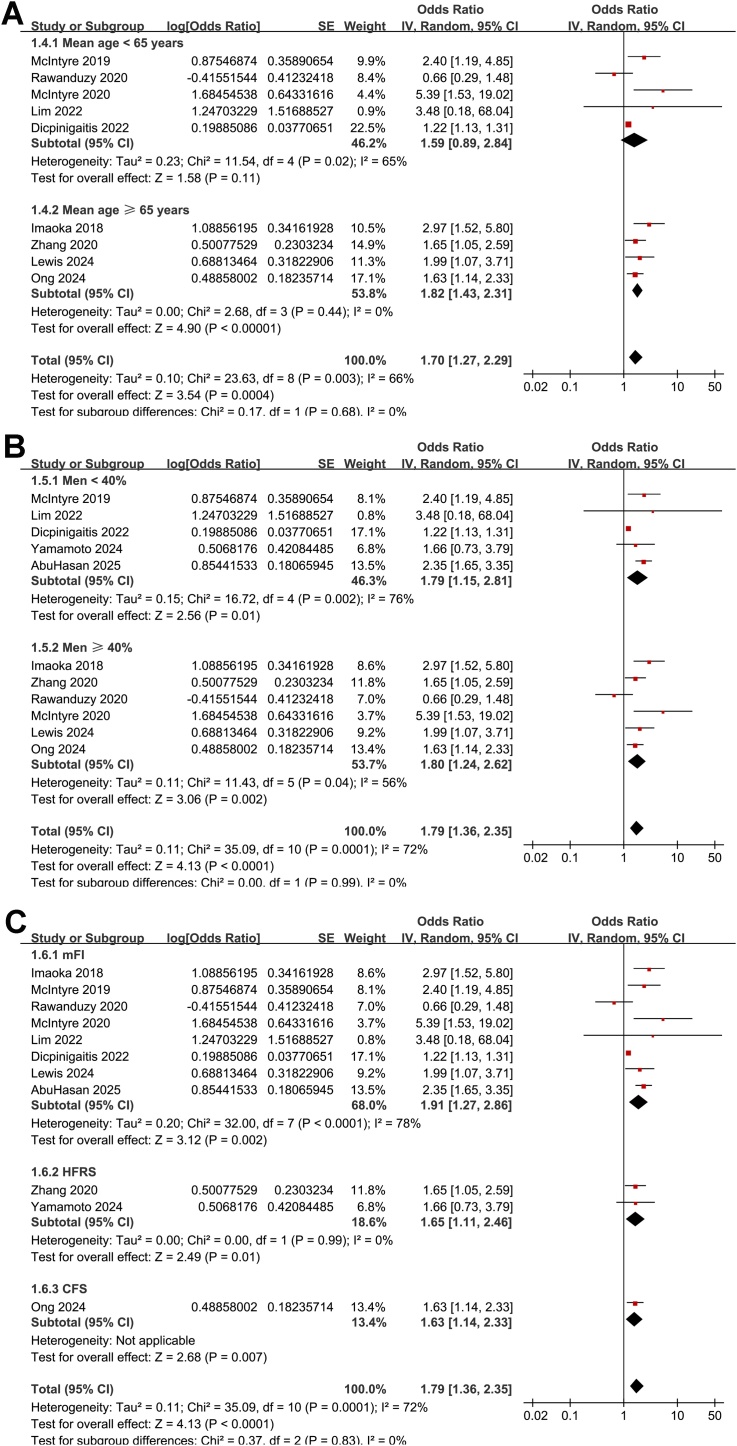
Forest plots for the subgroup-analysis of the association between frailty and short-term mortality of patients with ICrH. A, subgroup analysis according to the mean age of the patients; B, subgroup analysis according to the proportion of men; and C, subgroup analysis according to the methods for evaluating frailty; All estimates are adjusted ORs derived from multivariable analyses.

**Fig. 4:**
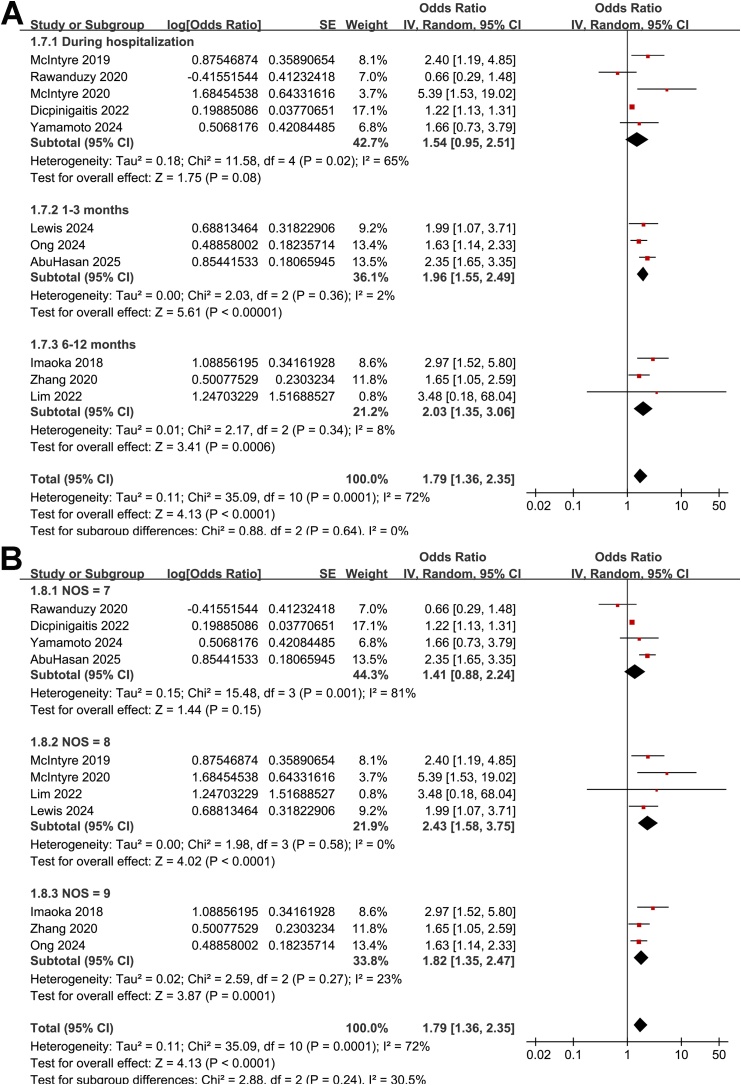
Forest plots for the subgroup-analysis of the association between frailty and short-term mortality of patients with ICrH. A, subgroup analysis according to the mean follow-up durations; and B, subgroup analysis according to the study quality scores; All estimates are adjusted ORs derived from multivariable analyses.

The meta-analysis of the 11 cohort studies [[20], [21], [22], [23], [24], [25], [26], [27], [28], [29], [30]] showed that frailty was associated with an increased risk of short-term mortality of patients with ICrH (OR: 1.79, 95% CI: 1.36–2.35, *p* < 0.001; Fig. 2A) with moderate heterogeneity (I^2^ = 72%). The sensitivity analysis was performed by omitting one dataset at a time, which did not significantly affect the results (OR: 1.69–1.92, *p* all < 0.05). Subsequent subgroup analyses showed that the association between frailty and increased risk of mortality in patients with ICrH was consistent in studies from Asian and Western countries (*p* for subgroup difference = 0.77, Fig. 2B), in patients with ICH and SAH (*p* for subgroup difference = 0.71, Fig. 2C), in patients of mean ages < and ≥65 years (*p* for subgroup difference = 0.68, Fig. 3A), in cohorts of the proportions of men < and ≥40% (*p* for subgroup difference = 0.99, Fig. 3B), and in studies with frailty evaluated by mFI, HFRS, and CFS (*p* for subgroup difference = 0.83, Fig. 3C). In addition, further subgroup analyses showed similar results in studies within follow-up during hospitalization, within 1–3 months, and 6–12 months after ICH onset (*p* for subgroup difference = 0.64, Fig. 4A), and in studies with NOS scores of seven, eight, and nine (*p* for subgroup difference = 0.24, Fig. 4B).

**Fig. 5:**
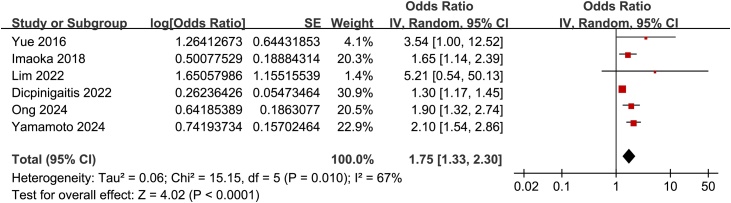
Forest plots for the meta-analysis of the association between frailty and the risk of poor functional outcome of patients with ICrH; All estimates are adjusted ORs derived from multivariable analyses.

Further meta-analysis with the six studies [19,20,24,26,29,30] showed that frailty was also associated with a poor functional outcome of patients with ICrH (OR: 1.75, 95% CI: 1.33–2.30, *p* < 0.001; Fig. 5) with moderate heterogeneity (I^2^ = 67%). Subsequent sensitivity analysis by excluding one study at a time showed similar results (HR: 1.62–1.95, *p* all < 0.05).

### 3.4 Publication bias

**Fig. 6:**
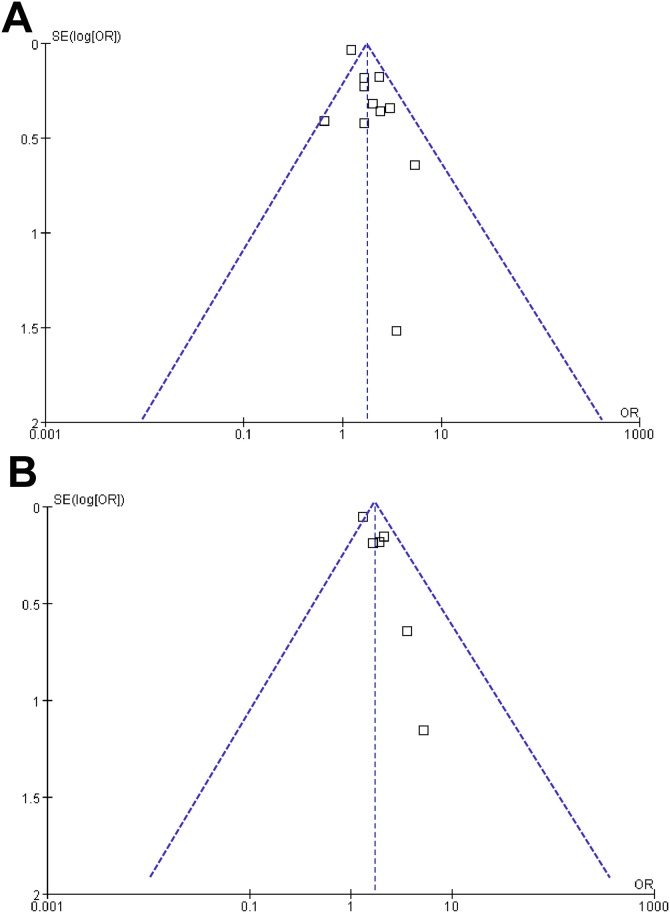
Funnel plots for meta-analysis of the associations between frailty and short-term prognosis of patients with ICrH; A, funnel plots for the meta-analysis of the association between frailty and short-term mortality of patients with ICrH; and B, funnel plots for the meta-analysis of the association between frailty and the risk of poor functional outcome of patients with ICrH;

Visual inspection of the funnel plots for the meta-analyses examining the association between frailty and all-cause mortality and poor functional outcome of patients with ICrH showed symmetry, suggesting a low likelihood of publication bias (Fig. 6A and B). Furthermore, the Egger’s regression test for the outcome of mortality also indicated a low risk of publication bias (*p* = 0.22). The Egger’s regression test was not performed for the incidence of poor functional outcome because only six studies were included.

## 4 Discussion

This meta-analysis provides comprehensive evidence that frailty is an independent predictor of short-term mortality and poor functional outcomes in patients with ICrH. By synthesizing data from twelve cohort studies involving 70,664 patients, we found that frail individuals had a significantly higher risk of all-cause mortality and poor functional recovery compared to non-frail counterparts. The association between frailty and mortality remained robust across multiple subgroup and sensitivity analyses, underscoring the consistency of this relationship. These findings emphasize the prognostic significance of frailty in ICrH and highlight the importance of incorporating frailty assessment into clinical practice to improve risk stratification and management strategies.

Several pathophysiological and clinical mechanisms may explain the link between frailty and poor prognosis in ICrH. Frail individuals often exhibit systemic inflammation, endothelial dysfunction, oxidative stress, and impaired neurovascular regulation [37,38], which contribute to increased vulnerability to cerebrovascular insults. These factors can exacerbate secondary brain injury following ICrH by promoting blood-brain barrier disruption, neuroinflammation, and metabolic stress, ultimately worsening neurological recovery [39,40]. In addition, frailty is frequently associated with sarcopenia, poor nutritional status, immunosenescence, and reduced physiological reserve [[41], [42], [43]], which impair the ability to withstand acute illness, delay recovery, and increase susceptibility to complications such as infections and prolonged immobilization [44]. Clinically, frail patients may have more comorbidity, lower functional reserves, and a higher likelihood of treatment complications [45,46], all of which contribute to worse survival and functional outcomes after ICrH. These combined pathophysiological and clinical factors likely explain why frailty is a strong predictor of both mortality and poor neurological recovery in patients with ICrH.

Subgroup analyses provided further insights into the relationship between frailty and short-term mortality in ICrH. The association between frailty and increased mortality was consistent across different geographical regions, subtypes of ICrH, patient age groups, sex distribution, frailty assessment methods, follow-up durations, and study quality scores. These findings suggest that frailty may represent a broadly applicable prognostic indicator of poor outcomes in ICrH, despite variations in patient characteristics, follow-up durations, and methods of frailty assessment. The absence of significant subgroup differences based on ICrH subtype indicates that frailty plays a comparable prognostic role in both ICH and SAH, despite their distinct pathophysiological mechanisms. Similarly, although the association between frailty and outcomes was consistent across different frailty assessment tools, the variability in both measurement approaches and follow-up durations across studies may have contributed to residual heterogeneity. Sensitivity analyses confirmed the robustness of our findings, as the exclusion of individual studies did not significantly alter the overall results. These consistent findings strengthen the validity of our conclusions and highlight the importance of frailty assessment in clinical practice.

This study has several strengths that enhance its credibility. First, we exclusively included cohort studies, which could therefore determine a longitudinal relationship between frailty and poor outcome of patients with ICrH. In addition, we conducted multiple subgroup and sensitivity analyses, which consistently supported the robustness of our findings. The inclusion of only multivariate-adjusted data strengthens the validity of the association between frailty and poor prognosis in ICrH, minimizing the influence of potential confounders. Collectively, these strengths make our study one of the most rigorous and comprehensive evaluations of the impact of frailty on ICrH outcomes. However, several limitations should be acknowledged. Most of the included studies were retrospective cohort studies, which are inherently prone to recall and selection biases [47]. Additionally, frailty assessment was not uniform across studies. While our subgroup analyses showed no significant differences between these tools, variability in frailty assessment may still contribute to heterogeneity. Moreover, the timing of frailty assessment was not clearly specified in most of the included studies, particularly the retrospective ones. Only one prospective cohort study evaluated frailty based on data from hospitalizations preceding the ICrH event [23]. Therefore, it remains uncertain whether frailty was assessed before or after the onset of ICrH in many studies, which may affect the interpretation of frailty as a pre-existing risk factor. Another limitation is the potential presence of unadjusted confounders. Although all included studies performed multivariate analyses, some key factors such as comorbidities, hemorrhage volume, and treatment strategies may not have been fully accounted for. Besides, while all included studies performed multivariable-adjusted analyses, the covariates included in these models varied widely across studies. This heterogeneity in statistical adjustment may have influenced the magnitude of the associations and contributed to between-study variability, which should be considered when interpreting the pooled results. Furthermore, our analysis was based on study-level data rather than individual patient data, preventing a more granular assessment of patient characteristics. This limitation also precluded the evaluation of certain factors, such as the impact of specific comorbidities and treatment approaches on the relationship between frailty and outcomes. Future research incorporating individual patient-level data could provide more precise risk estimates and allow for a more detailed exploration of modifying factors.

The findings of this study have important implications for clinical practice, particularly in nursing and patient management. Frailty assessment should be integrated into routine clinical evaluation for patients with ICrH to improve early risk stratification and guide treatment decisions. Early identification of frail patients may allow for more personalized care strategies, including intensive monitoring, early rehabilitation interventions, and targeted management of comorbidities [48]. In the nursing field, frailty screening can help inform tailored care plans, emphasizing early mobilization, nutritional support, and prevention of hospital-associated complications such as infections and pressure ulcers [49]. Given the significant impact of frailty on ICrH prognosis, healthcare teams should adopt a multidisciplinary approach, incorporating geriatric assessment and rehabilitation strategies to optimize outcomes in this vulnerable patient population. Future research should focus on standardizing frailty assessment tools for patients with ICrH and validating these tools in prospective studies. Additionally, studies with individual patient-level data are needed to better evaluate the role of comorbidities, hemorrhage characteristics, and treatment strategies in modifying the impact of frailty on prognosis. Further investigation into targeted interventions aimed at mitigating frailty-related risks, such as prehabilitation programs and post-acute rehabilitation strategies, may help improve outcomes in frail ICrH patients [50]. Exploring the impact of frailty on long-term neurological and functional recovery beyond the short-term follow-up period would also provide valuable insights.

## 5 Conclusions

In conclusion, this meta-analysis suggests that frailty may be associated with an increased risk of short-term mortality and poor functional outcomes in patients with ICrH. The association remains consistent across various patient demographics, frailty assessment tools, and study characteristics. These findings highlight the importance of frailty assessment in guiding risk stratification and clinical decision-making. Integrating frailty screening into routine practice may help optimize care strategies and improve outcomes for patients with ICrH. Future studies should aim to refine frailty assessment methods and explore interventions that can mitigate frailty-related risks in this patient population.

## Ethical standards

This review is based on existing literature. Therefore, this study did not include human participants or animals or require participant consent.

## Declaration of Generative AI and AI-assisted technologies in the writing process

Generative AI and AI-assisted technologies were not used in the writing process.

## Funding

No funding was received for this work.

## Declaration of competing interest

The authors declared no conflicts of interest.
