# Supplementary material for: Frailty and the short-term prognosis of patients with intracranial hemorrhage: A meta-analysis
Source: J Nutr Health Aging. 2025 Sep 9;29(10):100655. doi: 10.1016/j.jnha.2025.100655 (PMC12455095; doi:10.1016/j.jnha.2025.100655)
Supplement: Supplementary file 1 [file mmc1.docx]

**PubMed**

("Frailty"[Mesh] OR frailty OR frail)

AND

("Cerebral Hemorrhage"[Mesh] OR "Intracranial Hemorrhages"[Mesh] OR "Subarachnoid Hemorrhage"[Mesh]

OR "intracerebral hemorrhage" OR "brain hemorrhage" OR "intracranial hemorrhage" OR "ICH"

OR "cerebral bleeding" OR "intraparenchymal hemorrhage" OR "intracranial bleeding"

OR "subarachnoid hemorrhage" OR "subarachnoid haemorrhage" OR "SAH")

**Embase**

('frailty'/exp OR frailty OR frail)

AND

('brain hemorrhage'/exp OR 'intracerebral hemorrhage'/exp OR 'intracranial hemorrhage'/exp OR 'subarachnoid hemorrhage'/exp

OR 'cerebral hemorrhage' OR 'intracerebral hemorrhage' OR 'brain hemorrhage' OR 'intracranial hemorrhage' OR 'ICH'

OR 'cerebral bleeding' OR 'intraparenchymal hemorrhage' OR 'intracranial bleeding'

OR 'subarachnoid hemorrhage' OR 'subarachnoid haemorrhage' OR 'SAH')

**Web of Science**

TS=("frailty" OR "frail")

AND

TS=("cerebral hemorrhage" OR "intracerebral hemorrhage" OR "brain hemorrhage" OR "intracranial hemorrhage"

OR "ICH" OR "cerebral bleeding" OR "intraparenchymal hemorrhage" OR "intracranial bleeding"

OR "subarachnoid hemorrhage" OR "subarachnoid haemorrhage" OR "SAH")
